# Phenolic Compounds Inhibit 3T3-L1 Adipogenesis Depending on the Stage of Differentiation and Their Binding Affinity to PPARγ

**DOI:** 10.3390/molecules24061045

**Published:** 2019-03-16

**Authors:** Paula Aranaz, David Navarro-Herrera, María Zabala, Itziar Miguéliz, Ana Romo-Hualde, Miguel López-Yoldi, J. Alfredo Martínez, José Luis Vizmanos, Fermín I. Milagro, Carlos Javier González-Navarro

**Affiliations:** 1 Centre for Nutrition Research, University of Navarra, Irunlarrea 1, 31008 Pamplona, Spain; paranaz@unav.es (P.A.); dnherrera@alumni.unav.es (D.N.-H.); zabalan@unav.es (M.Z.); imigueliz@unav.es (I.M.); aromo@unav.es (A.R.-H.); mlyoldi@alumni.unav.es (M.L.-Y.); jalfmtz@unav.es (J.A.M.); fmilagro@unav.es (F.I.M.); 2 Department of Biochemistry and Genetics, University of Navarra, Irunlarrea 1, 31008 Pamplona, Spain; jlvizmanos@unav.es; 3 Department of Nutrition, Food Science and Physiology, University of Navarra, Irunlarrea 1, 31008 Pamplona, Spain; 4 Navarra Institute of Health Research (IdiSNA), 31008 Pamplona, Spain; 5 Spanish Biomedical Research Centre in Physiopathology of Obesity and Nutrition (CIBERObn); Instituto de Salud Carlos III, Monforte de Lemos 3-5, 28029 Madrid, Spain

**Keywords:** adipogenesis, adipocyte, 3T3-L1, polyphenol, phenolic acid, PPARγ, Nile Red

## Abstract

Phenolic compounds might modulate adiposity. Here, we report our observation that polyphenols and phenolic acids inhibit adipogenesis in 3T3-L1 with different intensity depending on the family and the stage of differentiation. While quercetin and resveratrol inhibited lipid accumulation along the whole process of differentiation, apigenin and myricetin were active during the early and latest stages, but not intermediate, contrary to hesperidin. The activity of phenolic acids was limited to the early stages of the differentiation process, except *p*-coumaric and ellagic acids. This anti-adipogenic effect was accompanied by down-regulation of *Scd1* and *Lpl*. Molecular docking analysis revealed that the inhibitory activity of these phenolic compounds over the early stages of adipogenesis exhibits a significant correlation (*r* = 0.7034; *p* = 0.005) with their binding affinity to the ligand-binding domain of PPARγ. Results show that polyphenols and phenolic acids would interact with specific residues of the receptor, which could determine their potential anti-adipogenic activity during the early stages of the differentiation. Residues Phe264, His266, Ile281, Cys285 and Met348 are the most frequently involved in these interactions, which might suggest a crucial role for these amino acids modulating the activity of the receptor. These data contribute to elucidate the possible mechanisms of phenolic compounds in the control of adipogenesis.

## 1. Introduction

At a cellular level, obesity is characterized by the increase in the number and size of adipocytes, which are differentiated from fibroblastic preadipocytes in adipose tissue [1]. Adipogenesis is a complex process that involves preadipocyte proliferation and adipocyte maturation in response to different stimuli and conditions [2]. This process is tightly regulated by sequential activations of various transcriptional factors, including CCAAT enhancer binding protein alpha (C/EBPα) and peroxisome proliferator-activated receptor gamma (PPAR)γ, which induce the expression of genes involved in lipid storage and contribute to the adipocyte maturation [2,3].

Based on its important role regulating both adipocyte differentiation and subsequent lipogenesis, the nuclear hormone receptor PPARγ has emerged as an interesting pharmacological target to control adipogenesis [4,5]. Structurally, this receptor contains six domains, although the ligand-binding domain (LBD) constitutes the major part of the protein and is responsible for the ability of PPARγ to direct adipogenesis and regulate insulin sensitivity [5]. In this sense, different molecules have emerged as biological ligands of PPARγ, interacting with variable affinity with the LBD and possibly modulating the activity of this receptor. Thus, these molecules have been proposed as a promising strategy to manage obesity and related diseases [6,7,8].

As an alternative, natural products are considered a potential pool of structures for drug discovery. There are different ongoing works aimed to explore the PPARγ-activating capability of a wide range of natural products, which could play a crucial role in the treatment or prevention of obesity-related diseases [8]. These compounds exhibit different molecular interactions with the LBD of PPARγ, which could be determined by their specific binding affinity to the receptor and therefore, their capacity modulating its activity [6,7,9,10,11]. 

Phenolic compounds have been widely studied during the last decade based on their antioxidant and anti-obesogenic properties. Thus, polyphenols such as resveratrol and curcumin have been the focus of different in vitro and in vivo assays to demonstrate their role in the inhibition of adipogenesis. Other studies have been performed with different phenolic compounds, such as flavonols, flavones and, less frequently, flavanones [12,13,14,15,16,17,18]. Some phenolic acids have also been proposed as potential anti-adipogenic agents [14,16,18,19,20,21,22,23]. Additionally, some of these phenolic compounds have been suggested as potential PPARγ ligands, including flavonoids (quercetin, kaempferol, luteolin), stilbenes (resveratrol) and phenolic acids (ellagic acid), based on their anti-obesogenic properties [8].

All these studies, mainly based on in vitro preadipocytic culture systems such as murine 3T3-L1 cells, have led to considerate polyphenols and phenolic acids as potential anti-adipogenic agents of relevance in the design of effective therapies to combat metabolic syndrome and related diseases. However, it is difficult to clearly assess the most effective anti-adipogenic compounds due to differences among the studies that hinder comparisons, as the studied compounds selected, the extent of treatment or the protocols used for the evaluation of their activity. Besides, the high doses used in some studies (above 100 µM) do not allow extrapolating the results to physiological conditions and, in some cases, this fact could lead to overestimations of the effect, based by masked cytotoxic side-effects. Finally, the underlying mechanisms of action of these compounds along the differentiation process are not completely understood, evidencing the hardness to determine if their action affects only the early stages of adipogenesis, or also the lipogenesis that accompanies the maturation of the already differentiated adipocyte. In this sense, during the last decade different reports have used molecular docking analysis to identify the possible interactions between ligands and receptors, which could help to understand the modulatory activity of these molecules [6,24].

Here, we investigate the effect of some different polyphenols (hesperidin, naringin, myricetin, quercetin, kaempferol, apigenin, luteolin, resveratrol and curcumin) and phenolic acids (*p*-coumaric, ellagic, ferulic, gallic and vanillic acids) on 3T3-L1 adipogenesis, when administered at non-cytotoxic doses at different points of the adipocyte differentiation process (early, intermediate and final stages). In addition, we have performed subsequent analyses of the underlying mechanisms involving their anti-adipogenic activity analysing the expression of some adipogenesis-key genes, such as *Pparg*, *Cebpa*, *Scd1*, *Fasn* and *Lpl*. Finally, due to the important role of PPARγ as master regulator of the pre-adipocytic differentiation into mature adipocyte [25], we have implemented an in silico molecular docking assay to determine the binding affinity of these phenolic compounds to the LBD of PPARγ. This latter has revealed differences for the binding specific site of each compound, identifying those specific residues that could help to explain the differences in the effectiveness of these molecules as potential anti-adipogenic agents.

## 2. Results and Discussion

The increasing prevalence of metabolic syndrome and related diseases, such as obesity and type-2 diabetes, is becoming one of the greatest public health problems of the 21st century [26]. In obesity, strategies for weight control management, including diet, exercise and modification of lifestyle are the basis of the current therapies. Additionally, the incorporation of natural bioactive compounds to the diet, including fatty acids, phenolic compounds, soybean, plant sterols, calcium, and dietary fibre is increasingly being considered a crucial strategy in the treatment and/or prevention of obesity and related diseases [27,28]. In this regard, one of the most promising mechanisms by which bioactive compounds counteract obesity is the inhibition of adipogenesis, either by blocking the initial preadipocytic differentiation, or by preventing the fat accumulation needed for the adipocyte maturation [29,30]. 

During the last decade, several studies have focused their efforts in the study of the anti-adipogenic activity of different natural bioactive compounds based on the murine 3T3-L1 in vitro model [2,3,31,32], including phenolic compounds present in natural sources primarily described as antioxidants [12,13,15,16,17,20,21,33,34,35]. 

Here, we investigated the potential anti-adipogenic effect of nine different polyphenols (apigenin, luteolin, hesperidin, naringin, resveratrol, curcumin, myricetin, kaempferol and quercetin) and five phenolic acids (*p*-coumaric, ellagic, ferulic, gallic and vanillic acids) along three different stages of the differentiation process in 3T3-L1 cells, and results were corrected for the possible cellular toxicity at the corresponding doses. Additionally, we analysed the expression of some key-genes involved in adipogenesis and performed an in silico molecular docking analysis to evaluate the possible role of these phenolic compounds as potential PPARγ modulators.

### 2.1. Phenolic Compounds Inhibited Adipogenesis with Different Intensity Depending on the Stage of the Differentiation Process

**Figure 1:**
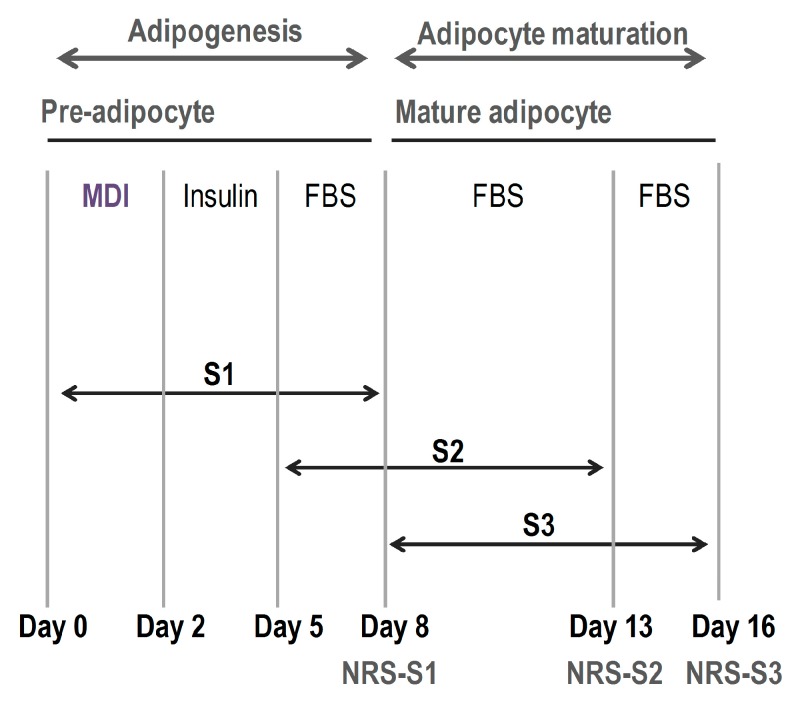
Scheme followed in the procedure to evaluate the effect of the treatments during different phases of the differentiation process: pre-differentiation/stage 1 (S1, from day 0 of the study to day 8), differentiation/stage2 (S2, from day 5 of the study to day 13) and post-differentiation/stage 3 (S3, from day 8 of the study to day 16), depending on the moment the compounds were added to the medium. Treatments were maintained during 8 days in every case, until Nile Red staining (NR) was performed at the three analytical points indicated (NRS-S1, NRS-S2 and NRS-S3).

In order to analyse the ability of these compounds to reduce the triglyceride content along the initial, intermediate and final stages of the differentiation process, all treatments were evaluated at three different points (S1, S2 and S3, respectively), depending on the moment when compounds were added to the medium (Figure 1). 

**Figure 2:**
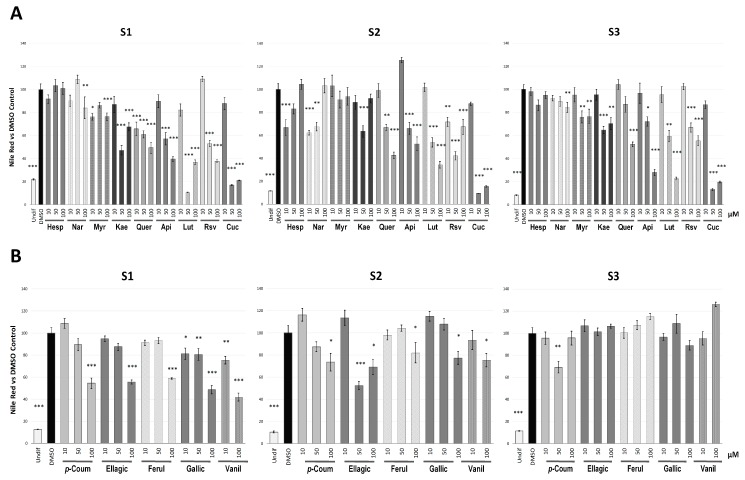
Quantification of the intracellular triglyceride content, measured by Nile red staining, of 3T3-L1 cells after treatment with polyphenols (**A**) and phenolic acids (**B**) along the three stages evaluated (S1, S2 and S3). Results expressed as the mean percentage (±SD) of cell triglyceride content relative to DMSO-treated control cells. Undifferentiated 3T3-L1 cells (Undif) were used as positive control. Significance refers to the effect of each treatment with respect to DMSO-control cells (* *p* < 0.05; ** *p* < 0.01; *** *p* < 0.001). Cells were collected on day 8, day 13 and day 16 for the S1, S2 and S3 analyses, respectively. Abbreviations: Hesp (hesperidin), Nar (naringin), Myr (myricetin), Kae (kaempferol), Quer (quercetin), Api (apigenin), Lut (luteolin), Rsv (resveratrol), Cur (curcumin), *p*-Coum (*p*-coumaric acid), Ellagic (ellagic acid), Ferul (ferulic acid), Gallic (gallic acid), Vanil (vanillic acid).

Nile Red results evidenced differences in the inhibitory ability of the polyphenols (Figure 2A) and phenolic acids (Figure 2B) among families and stages of the adipogenesis process, in comparison with DMSO-treated control cells. Figure S1 represents the cell imaging obtained by fluorescent microscopy after treatment of the different compounds (dose of 100 µM) in the S1 assay.

To avoid false positive results due to a putative cytotoxic effect of certain doses of these compounds, we evaluated the cell viability of 3T3-L1 pre-adipocytes 48 h after the different treatments in comparison with DMSO-treated control cells (Table S2). This evaluation determined that the lipid reduction induced by some treatments, such as luteolin, kaempferol and naringin, could not be certainly attributed to an anti-adipogenic activity, due to the effect of these compounds on 3T3-L1 cell viability. 

### 2.2. Quercetin, Resveratrol and Vanillic Acid were the Most Effective Phenolic Compounds Inhibiting Adipogenesis

**Figure 3:**
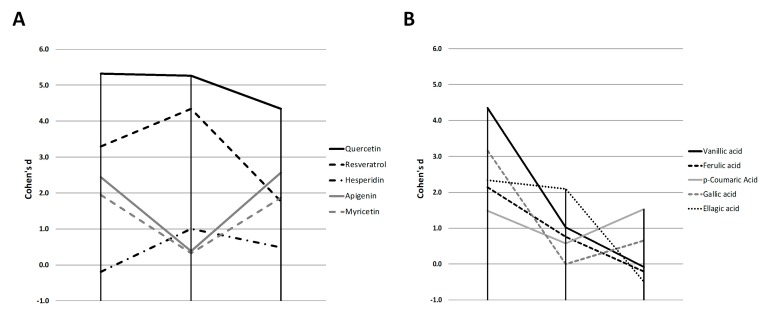
Representation of the Cohen’s D effect size for the anti-adipogenic activity of the different polyphenols (**A**) and phenolic acids (**B**) during the three evaluated stages (S1, S2 and S3). Those compounds with an observed toxicity at doses of 50 µM or lower were excluded.

With the objective of establishing a classification of the anti-adipogenic activity among compounds, we determined the Cohen’s d effect size for their ability to reduce the 3T3-L1 triglyceride content (based on NR data), corrected by the corresponding value of cell viability (in%) for each concentration (Table S3). The calculations were performed considering the average of the effect observed at all doses that did not induce a significant reduction of 3T3-L1 cell viability. The phenolic compounds exhibited different effect sizes depending on the stage (S1, S2 and S3) of the differentiation (Figure 3). 

Thus, apigenin, and myricetin showed a similar “V-shaped” profile along time, inhibiting triglyceride accumulation in S1 and S3 stages. They showed a lower activity when treated at day 5 of differentiation (S2), in comparison with the other flavonol quercetin (Figure 3A). Contrary to this result, resveratrol and hesperidin showed an inverted profile, with the strongest effect when added in the intermediate stages of the process (S2), suggesting a specific inhibitory activity at the intermediate step of adipogenesis. Finally, quercetin showed similar activity at both the initial and intermediate stages (S1 and S2) but it reduced its activity when the adipocyte had reached the mature stage of differentiation (S3), pointing that this compound is more effective during the early and intermediate stages of differentiation. Nevertheless, it is remarkable that quercetin activity, which did not show cytotoxicity on 3T3-L1, is the strongest and the most sustained all along the three stages.

For most phenolic acids (Figure 3B), the strongest effect size was observed in S1, being vanillic acid the most effective one. However, only ellagic acid and *p*-coumaric acid maintain the anti-adipogenic activity at the intermediate and final stages of differentiation, respectively. This may suggest that most phenolic acids assayed strongly inhibit the early stages of differentiation, but they do not maintain the anti-lipogenic activity in mature adipocytes. The observed activity of *p*-coumaric acid in S3 could prompt a specific activity of this phenolic acid, more similar to polyphenols such as apigenin, myricetin or quercetin.

These results are consistent with other studies previously reported in the literature. Hsu and collaborators evaluated the capacity for inhibiting adipogenesis of a wide range of phenolic compounds and six flavonoids in 3T3-L1 cells [18]. In this study, which did not include an analysis of cell viability, treatments were performed at day 8 of differentiation (our S3) with different doses (0–250 µM) for 72 h and 3T3-L1 differentiation rate was determined by ORO staining. Results showed that rutin, the quercetin rutinoside, was the flavonoid with the highest inhibitory capacity (83%), followed by quercetin (44.6%), vanillic acid (43.5%), resveratrol (41.8%), and hesperidin (40.2%). Concerning phenolic acids, the study of Hsu and Yen reported that vanillic acid exhibited the strongest effect (43.5%), followed by *p*-coumaric acid (31.4%), gallic acid (29.2%) and ferulic acid (16.8%), with the lower inhibitory efficiency [18]. Our results for vanillic acid also show the highest activity at S1 stage but, interestingly, one of the lowest at S3 stage. Consistently with our results, other work showed that ferulic acid also exhibited the lower activity in reducing the intracellular triglyceride content of 3T3-L1 preadipocytes, in comparison with other phenolic acids [22]. However, other studies performed with phenolic acids have suggested that their potential anti-adipogenic effect is restricted to the early stages of differentiation [20,22,35]. Finally, a study combining different times of treatment demonstrated that p-coumaric acid (100 µM) exerted anti-adipogenic effects on 3T3-L1 adipocytes when added during our defined S1 stage, and at a late stage of differentiation [23]. This result agrees with our observation that p-coumaric was the only phenolic acid with significant reduction of differentiation in S3 assay.

### 2.3. Phenolic Compounds Block the Early Stages of Adipogenesis by Regulating the Expression of Adipogenic Genes

At molecular level, it has been described that, upon the addition of the adipogenic cocktail (including insulin, dexamethasone and IBMX) to 3T3-L1 preadipocytes, there is a rapid and transient elevation in the transcription factors C/EBPβ and C/EBPσ, which induce the activation of the nuclear hormone receptor peroxisome proliferator-activated receptor γ (PPARγ) and C/EBPα [36]. These two latter remain elevated for the rest of the differentiation process and indeed for the life of the mature adipocyte. In fact, these factors control the expression of adipocyte-specific genes, such as *Fasn*, *Lpl* or *Scd1*, which constitute key regulators of the fatty acid metabolism, triglyceride uptake and lipid storage [2,3,37,38,39].

**Figure 4:**
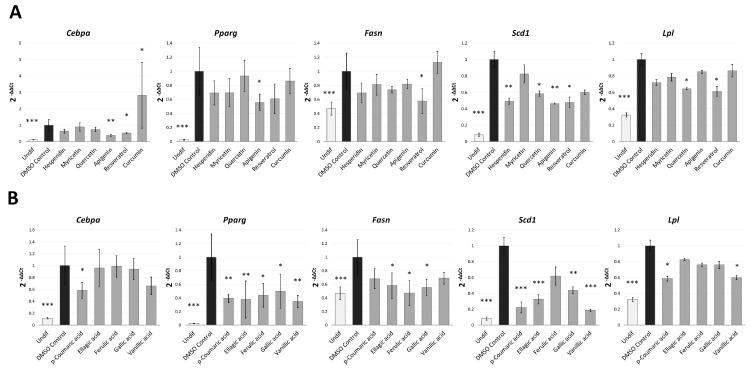
Gene expression analysis of adipogenesis-related genes after treatment (20 µM) with the different polyphenols (**A**) and phenolic acids (**B**) in the S1 analysis (cells collected on day 8). Results are expressed as the fold-difference expression levels of each gene in the treated group compared with the DMSO-control cells, calculated with the 2^−∆∆Ct^ method. Significance refers to the effect of each treatment with respect to DMSO-control cells (* *p* < 0.05; ** *p* < 0.01; *** *p* < 0.001).

Our Nile Red results suggested that the anti-adipogenic activity of the phenolic compounds analysed could be mainly attributed to the inhibition of the early stages of the preadipocyte differentiation, when the machinery of adipogenesis becomes activated. For this reason, in order to clarify the molecular effect of these compounds during the S1 stage we analysed by real-time PCR (qPCR) the gene expression levels of the main adipogenesis-related transcription factors (PPARγ and CEBPα) and some adipocyte-specific genes (*Scd1*, *Fasn* and *Lpl*) after treatments at the dose of 20 µM, as the 2-fold the minimum assayed dose (10µM) used in the efficacy assay (Figure 4). 

The same dose was used for all compounds in order to determine the most effective treatment at a common concentration. Those treatments that induced a significant effect on cell viability at the dose of 10 µM (kaempferol, luteolin and naringin, Table S2) were excluded of this analysis. As expected, untreated 3T3-L1 preadipocytes, showed a significantly reduced gene expression of the transcription factors PPARγ (*p* < 0.001) and CEBPα (*p* < 0.001), and the genes *Fasn* (*p* = 0.006), *Scd1* (*p* < 0.001) and *Lpl* (*p* < 0.001) in comparison with DMSO-control cells.

In the case of polyphenols (Figure 4A), resveratrol treatment showed the more widespread effect on gene expression, as it down-regulated the expression of the transcription factor CEBPα (*p* = 0.012), and the adipocyte-specific genes *Fasn* (*p* = 0.017), *Lpl* (*p* = 0.016) and *Scd1* (*p* = 0.030). The protein SCD-1 constitutes an important regulator of leptin activity and is the rate-limiting enzyme in monounsaturated fatty acid biosynthesis [40]. Reduced SCD-1 activity may help to prevent obesity by suppressing fatty acid biosynthesis and activating fatty acid oxidation [40]. FASN is a terminal marker of adipocyte differentiation involved in fatty acid biosynthesis [38], so its expression is related to a lipogenic state in the cell. The lipoprotein lipase (LPL) is a central protein for successful adipogenesis and contributes to maintain adipose tissue. This protein plays an important role in lipid uptake and utilization by various cell types [37,38,39,41]. Thus, down-regulation of *Cebpa*, *Scd1*, *Fasn* and *Lpl* by resveratrol treatment demonstrates the potent inhibitory activity of this polyphenol on both adipogenesis and lipogenesis processes. This result is consistent with other works [29,38,42], and could explain the strong lipid reduction observed along the whole process of differentiation. Although it has been reported that resveratrol treatment can inhibit *Pparg* gene expression [29,43], we could not detect this effect (*p* = 0.125) at the dose assayed (20 µM).

Apigenin was the only polyphenol able to significantly reduce *Pparg* (*p* = 0.021) gene expression. This flavone also markedly down-regulated *Cebpa* (*p* = 0.003) and *Scd1* (*p* = 0.002), remarking the important anti-adipogenic effect of this compound at the early stages of differentiation. Consistent with this result, a previous study showed that apigenin (0–50 µM) suppressed 3T3-L1 adipocyte differentiation and reduced intracellular lipid accumulation (quantified by Oil Red O staining), through the down-regulation of *Pparg* and its target genes *Fabp4* and *Scd1* [33]. 

Hesperidin and quercetin treatments also reduced *Scd1* gene expression (*p* = 0.005 and *p* = 0.029, respectively), demonstrating the anti-lipogenic capacity of these compounds. In addition, quercetin treatment down-regulated *Lpl* (*p* = 0.040), contributing to explain the inhibitory activity of this flavonoid along the differentiation. Myricetin, a flanovol with a similar structure to quercetin but with lower anti-adipogenic activity (measured by NR), did not affect gene expression significantly. Remarkably, curcumin, which exhibited a high toxicity effect from the dose of 50 µM, did not show a relevant effect on the expression of adipogenic genes. In fact, this compound significantly upregulated *Cebpa* (*p* = 0.022). This data supports the hypothesis that the strong effect of curcumin on the intracellular lipid accumulation observed (Figure 2) could be mainly attributed to their cytotoxic effect at the studied dose and not to an inhibitory activity over adipogenesis.

In the case of phenolic acids (Figure 4B), the five compounds were found to down-regulate *Pparg* (*p* < 0.05). Except in the case of ferulic acid, one of the phenolic acids with lowest effect on intracellular lipid accumulation, this effect was accompanied by a significant reduction of *Scd1* (*p* < 0.05). Moreover, ellagic, ferulic and gallic acids down-regulated the lipogenic gene *Fasn* (*p* = 0.038, *p* = 0.018 and *p* = 0.021, respectively), while, *p*-coumaric and vanillic acids significantly reduced the expression of *Lpl* (*p* = 0.014 and *p* = 0.026, respectively), involved in lipid uptake and lipogenesis [37,41]. Similarly to resveratrol and apigenin, only p-coumaric acid was able to down-regulate *Cebpa* (*p* = 0.048), which could explain the inhibitory effect of this phenolic acid along both the S1 and S3 stages of differentiation, unlike the rest of phenolic acids, which were not effective at the latest stages of the process.

### 2.4. Molecular Docking Analysis Reveals that PPARγ Interaction is Crucial for the Inhibition of Adipogenesis

In the early stages of adipogenic commitment, the nuclear receptor PPARγ is activated and, together with its primary target C/EBPα, induces adipogenesis through the activation of different adipocyte specific genes needed for triglyceride uptake and lipid storage. PPARγ is therefore considered a master regulator of adipogenesis, although it also plays an important role in insulin sensitivity and glucose homeostasis and modulates metabolism and inflammation [5,8]. Thus, PPARγ has emerged as an interesting pharmacological target and the modulation of its activity might be an effective strategy to regulate obesity and related diseases. Different molecules have been suggested as biological ligands of PPARγ, such as Troglitazone [44], used to combat hyperglycaemia associated with the metabolic syndrome and type-2 diabetes. In this sense, natural products have proven historically to be a promising pool of structures for drug discovery. Thus, some ongoing works are exploring the PPARγ-activating potential of a wide range of natural products, which could play a crucial role in the treatment or prevention of obesity-related diseases. These putative modulators show several molecular interactions with the LBD of PPARγ, which could determine their binding affinity (kcal/mol) to this protein and thus, their modulating capacity [6,9,10].

On the other hand, over the last years the use of bioinformatics tools such as molecular docking has become an essential part of research, focused at prediction of the binding of small molecules to their target proteins [45]. In this work, we performed an in silico molecular docking analysis to determine whether the inhibitory activity of the phenolic compounds observed during the early stages of differentiation could be explained by their ability to modulate the PPARγ activity, providing a mechanistic insight into the effects of these compounds on adipogenesis. The 3D structures of 14 putative ligands (9 polyphenols and 5 phenolic acids) were tested to compare their predicted interaction to PPARγ receptor, as well as to describe the major amino acid residues of the ligand-binding domain (LBD) of the protein involved in these interactions. 

The analysis was performed using DockingServer (www.dockingserver.com), a web-based interface which allows performing molecular docking using AutoDock tools [45,46]. The values of free energy of binding (kcal/mol), inhibition constant (Ki), total estimated energy of dcW + Hbond + desolv (EVHD), total intermolecular energy, frequency of binding, and interact surface area were evaluated and rank the compounds by their estimated free energy of binding (kcal/mol) to the protein (Table 1) [24,45]. 

Binding energies are representative of how precisely the ligand (phenolic natural products or drug candidates) binds to the target protein, where lower (more negative) energy values correspond to more favourable ligand binding, while higher energy (closer to 0) values are less favourable [24,45,47,48]. This analysis revealed that the different compounds analysed exhibit different binding affinities to PPARγ chains A and B, using troglitazone as reference. For the most favourable binding of troglitazone, estimated free energy of binding was −7.23 kcal/mol, with a total intermolecular energy of −8.93 kcal/mol. The flavones luteolin and apigenin exhibited the lowest free energy of binding, which means the highest binding affinity score. This is consistent with a previous work that reported that the anti-adipogenic effects of luteolin were mediated by transactivation of PPARγ [49]. In comparison, the flavonols kaempferol and quercetin exhibited a free energy of binding of −5.27 and −5.07 kcal/mol, respectively, showing higher affinities to interact with PPARγ than myricetin (−4.34 kcal/mol).

**Figure 5:**
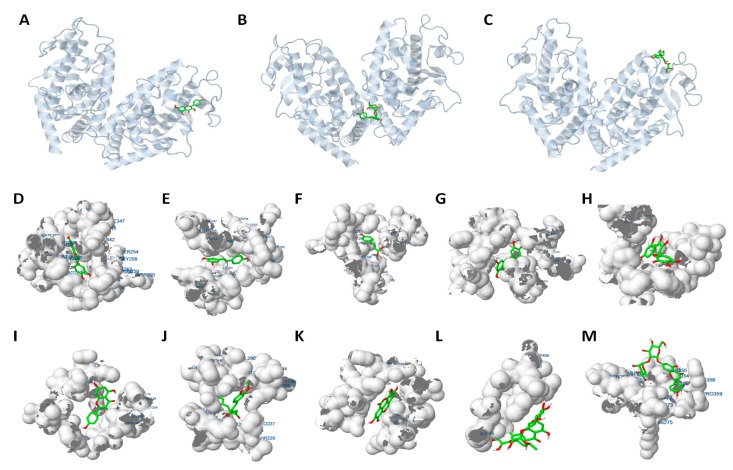
Representation of the specific interaction of different phenolic compounds with PPARγ. (**A**–**C**) Location of the PPARγ binding site for quercetin (**A**), hesperidin (**B**) and naringin (**C**). PPARγ structure (grey) is represented in cartoon and phenolic compounds (green and red) as bonded. (**D**–**M**) Closer view of phenolic compounds (bonded) interaction with the PPARγ ligand-binding domain (LBD, represented as electronic cloud surface, grey). Interaction with the LBD of quercetin (**D**), resveratrol (**E**), p-coumaric acid (**F**), luteolin (**G**), myricetin (**H**), kaempferol (**I**), curcumin (**J**), ellagic acid (**K**), hesperidin (**L**) and naringin (**M**).

Resveratrol and curcumin also showed a high binding affinity to PPARγ. It has been previously proposed that resveratrol could act as a PPARγ antagonist through its interaction with the receptor [50]. Hesperidin and naringin exhibited the highest values of energy of binding, demonstrating the poor binding affinity of these flavanones to PPARγ, and consistent with the fact that the former shows the lowest antiadipogenic effect, particularly at the S1 stage. In the case of phenolic acids, the highest binding affinity is observed for the ellagic acid (−5.64 kcal/mol). On the contrary, ferulic acid was the phenolic acid with the highest value of free energy of binding, which correlates with the low anti-adipogenic activity of this compound. The difference in the binding affinities of the phenolic compounds could be attributed to small differences in their structures, or to different binding sites of each molecule with the PPARγ receptor. The binding site of resveratrol, *p*-coumaric acid, luteolin, myricetin, kaempferol, curcumin and ellagic acid is located in the same protein region as quercetin (Figure 5A). The analysis shows that these compounds are also embedded in the LBD of this protein (Figure 5D–K). By contrast, consistent with their low binding affinity, the binding interactions of the flavanones hesperidin (Figure 5B) and naringin (Figure 5C) to the receptor occurs in a different region of the rest of compounds, and externally to the ligand binding domain (Figure 5M,N). The different types of interactions induced by each putative ligand with PPARγ, including the corresponding amino acids involved in the binding pocket of the receptor, and the type of atom of the phenolic compounds that binds with the residue are collected in Table S4. Figure S2 represents the 2D structure of the phenolic compounds of the study and putative hydrogens involved in *H*-interactions. Due to the similarity among their structures, the specific PPARγ residues of the LBD involved in each interaction may explain the differences in the binding affinities among compounds. 

**Figure 6:**
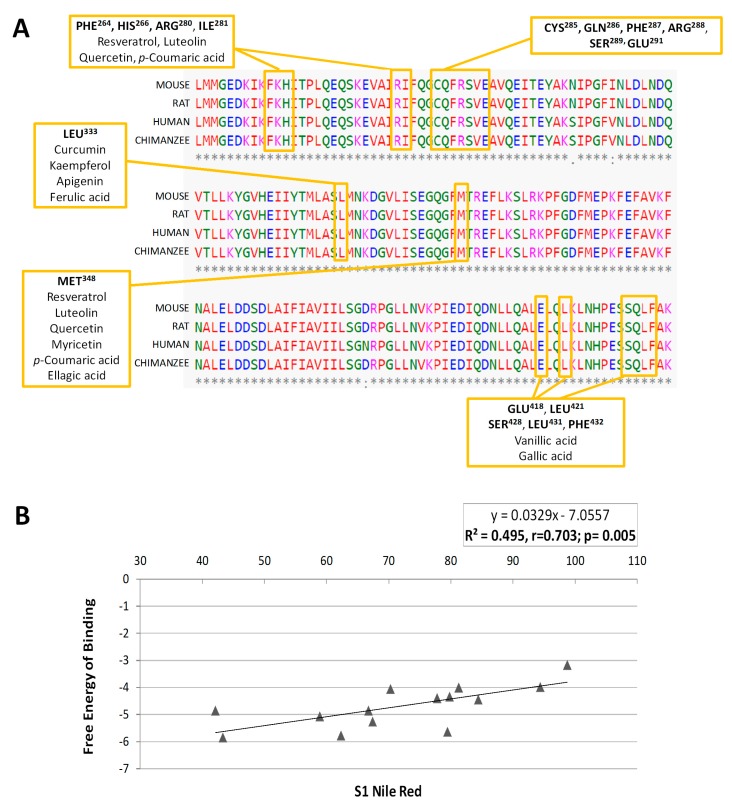
(**A**) Protein sequence of the PPARγ LBD implicated in the interactions with the phenolic compounds. The amino acid sequence is highly conserved between human and rat, mouse and chimpanzee proteins. The text boxes indicate the main amino acid residues with which the different phenolic compounds interact. (**B**) Representation of the Pearson correlation analysis between the inhibitory activity on 3T3-L1 adipogenesis in the S1 assay (quantified by Nile Red staining) and the free energy of binding (kcal/mol) for each compound to PPARγ protein.

Thus, the residues affected by the interactions are not the same for every phenolic compound, although there are some common amino acids, which would constitute the putative binding pocket of PPARγ (Table S4). The residues more frequently affected by the different treatments are indicated in Table 2. The amino acids Phe264, His266, Arg280, Ile281, Cys285 and Arg288 constitute the main residues involved in the interactions of PPARγ with the phenolic compounds analysed, together with Leu333 and Met348. These amino acids were found to constitute the binding site of PPARγ [6,10] and are very well conserved among species (Figure 6A), especially those amino acids shared by various compounds (highlighted). This is the case of resveratrol, quercetin, p-coumaric acid and luteolin, which bind the same residues of the protein. 

It is remarkable that, despite the similar structure of these compounds (Figure S2), they show different interactions. Thus, while quercetin and myricetin share interactions with the residues Glu259, Phe264, Cys285 and Met348, myricetin does not bind His266, Arg280 and Ile281. This data suggest that these three amino acids might play a critical and specific role in the PPARγ LBD, since compounds binding these residues (quercetin, resveratrol, luteolin and p-coumaric acid) show a higher affinity for the receptor. This fact might explain the lower inhibitory activity of myricetin on 3T3-L1 adipogenesis. 

On the contrary, kaempferol exhibits a particular binding pattern (residues Cys285, Arg288 and Leu333), different from the other studied flavonols, but similar to curcumin. This specific interaction pattern could be related to their higher cytotoxic effects in vitro. On the other hand, the flavones apigenin and luteolin, which exhibited the best binding energy values, showed a completely different binding pattern. While luteolin reminds quercetin, resveratrol or *p*-coumaric acid, apigenin shares some amino acids with ferulic acid (the phenolic acid with the lowest binding affinity). However, as in the case of kaempferol and curcumin, apigenin also binds to Cys285 and Arg288, pointing to a key role of these amino acids in the binding pocket of the receptor. This difference might explain both the different binding affinities and adipogenesis-inhibitory activities observed for apigenin and ferulic acid. 

The flavanones hesperidin and naringin, on the contrary, do not bind to any of the common residues described above (Table S4). This could explain their low binding affinity for the PPARγ receptor in comparison with the rest of polyphenols, and might be related to the specific anti-adipogenic activity of hesperidin, focused to the S2 stage of the differentiation. It is noticeable that, to differ from the rest of the tested compounds, hesperidin and naringin constitute the glycoside forms of their corresponding aglycons, hesperetin and naringenin, respectively. With the aim of determining whether the presence of the disaccharide could be affecting our results, we performed the docking analysis with the two corresponding aglycons. Thus, we showed that the binding affinity for hesperetin and naringenin are two of the strongest among compounds (Table 1), suggesting that the low affinity of hesperidin and naringin might be attributed to the presence of the glycoside. Additionally, the residues implied in the interactions of PPARγ with hesperetin and naringenin are also different to hesperidin and naringin, being located in the ligand-binding domain of the receptor and coinciding with those affected by other polyphenols (Table 2). Although there are differences in the amino acids affected by these flavanones, both of them bind the Glu291, Leu333 and Ile341, but not the Cys285, involved in the interaction with the rest of polyphenols, suggesting a specific binding-site for these two molecules. 

On the other hand, gallic and vanillic acid seems to share affinity by the same residues, Glu418, Leu421, Ser428, Leu431 and Phe432, very different to ellagic acid (more similar to curcumin and kaempferol), and p-coumaric acid (similar to resveratrol and quercetin). Interestingly, *p*-coumaric acid exhibited a different inhibitory activity on 3T3-L1 adipogenesis, inducing efficient lipid reduction not only in the early stages of differentiation (S1), but also in the latest ones (S3), similarly to some polyphenols. In fact, a study using computational biology tools demonstrated that *p*-coumaric acid (100 and 200 µM) inhibited 3T3-L1 lipid accumulation and could induce conformational changes in Pparγ2 [31].

**Figure 7:**
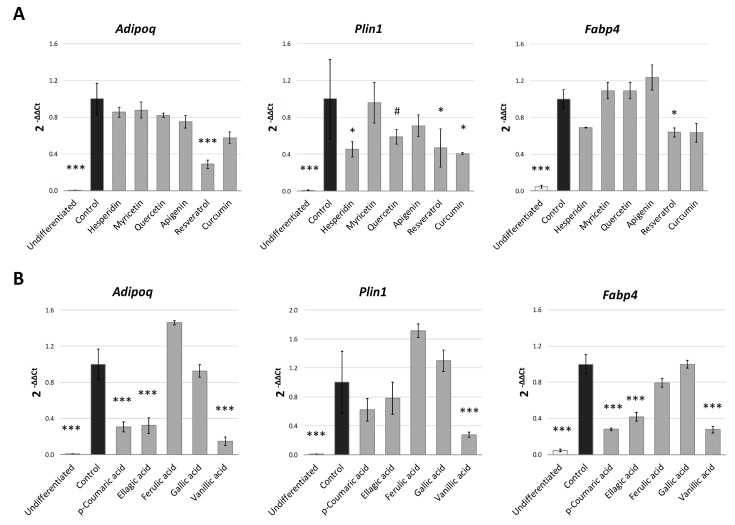
Gene expression analysis of PPARγ-target genes after treatment (20 µM) with the different polyphenols (**A**) and phenolic acids (**B**). Results are expressed as the fold-difference expression levels of each gene in the treated group compared with the DMSO-control cells, calculated with the 2^−∆∆Ct^ method. Significance refers to the effect of each treatment with respect to DMSO-control cells (* *p* < 0.05; ** *p* < 0.01; *** *p* < 0.001).

Finally, in order to verify whether the differences in the PPARγ binding affinity for each phenolic compound could explain the variation in their anti-adipogenic activity in 3T3-L1, we performed a Pearson’s correlation analysis between the fat accumulation effect (quantified by Nile Red) in S1 and the free-energy of binding values obtained for each compound (Figure 6B). This analysis showed a highly significant correlation (*r* = 0.7034; *p* = 0.005) between both values, suggesting that the potential inhibitory activity of the phenolic compounds over the early stages of adipogenesis in 3T3-L1 adipocytes could be, at least partially, explained by their capacity to interact with the binding pocket of the adipogenesis-mediator PPARγ. The changes found in the expression of genes regulated by PPARγ during adipogenesis, like *Scd1* [51] or *Lpl* [52] after treatment with phenolic compounds, would support our hypothesis. However, the use of *in silico* molecular modeling predictions for the explanation of a biological effect presents limitations. For this reason, we evaluated the expression of some additional adipocyte-specific genes that are also regulated by PPARγ. As it can be observed in Figure 7A, most polyphenols were able to reduce Plin1 expression, with the exception of myricetin, one of the flavonols with the lower affinity to PPARγ and reduced anti-adipogenic activity during S1 stage (Figure 3). Perilipin 1 (*Plin1*) is a lipid droplet-associated protein, which coats the surface of lipid droplets during adipogenesis [53,54], and its expression in adipocytes is controlled by PPARγ [55]. Additionally, and consisting with the previous qPCR data, resveratrol, a polyphenol with high effect during S1 stage (Figure 3), was the one with the strongest effect on other PPARγ-target genes, as it down-regulates *Adipoq* (*p* < 0.001) and *Fabp4* (*p* = 0.047). Adiponectin (*Adipoq*) is mainly produced by adipocytes during cell differentiation, increasing lipid content and insulin responsiveness of the glucose transport system in adipocytes [56,57], and its expression is controlled by PPARγ [58]. *Fabp4* encodes for the adipose-specific fatty acid binding protein aP2/FABP4, which plays important roles in fatty acid uptake, transport, and metabolism in adipocytes [59,60]. Despite the lack of significance, it is remarkable that most polyphenols induced a reduction in *Adipoq* gene expression of almost 20% with respect to DMSO-treated cells.

In the case of phenolic acids (Figure 7B), *p*-coumaric, ellagic and vanillic acids strongly reduced the *Adipoq* and *Fabp4* gene expression (*p* < 0.001), consisting with our previous data, where these compounds were more effective than ferulic and gallic acids binding PPARγ-LBD. Vanillic, the phenolic acid with the strogest effect on triglyceride accumulation (quantified by Nile red, Figure 3), was also able to reduce *Plin1* gene expression (*p* < 0.001), similarly to polyphenols. Thus, these results show a relationship between the effect during S1 stage, the in silico binding affinity to PPARγ and the expression of PPARγ-controlled genes related to adipogenesis.

## 3. Materials and Methods

### 3.1. Reagents

Chemicals were obtained from Sigma Aldrich (St. Louis, MO, USA): apigenin ≥97% (TLC), from parsley, powder, (ref. #A3145; PubChem CID: 5280443); luteolin analytical standard, ≥97.0% (HPLC, ref. # 72511; PubChem CID: 5280445); resveratrol ≥99% (HPLC, ref #R5010; PubChem CID: 445154); curcumin from *Curcuma longa* (*Turmerie*) powder (ref. #C1386; PubChem CID: 969516); kaempferol ≥90% (HPLC, ref. # K0133; PubChem CID: 5280863); quercetin ≥95% (HPLC, ref. #Q4951; PubChem CID: 5280343); myricetin analytical standard, ≥98% (HPLC, ref. #72576; PubChem CID: 5281672); hesperidin analytical standard, ≥97.0% (HPLC, ref. # 50162; PubChem CID: 10621); naringin ≥95% (HPLC, ref. #71162; PubChem CID: 442428); *p*-coumaric acid ≥98.0% (HPLC, ref. #C9008; PubChem CID: 637542); ellagic acid powder from tree bark ≥95% (HPLC, ref. #E2250; PubChem CID: 5281855); *trans*-ferulic acid >99%, (ref. #128708; PubChem CID: 445858); Gallic acid 97.5–102.5% (titration), (ref. #G7384; PubChem CID: 370); vanillic acid purum ≥97.0% (HPLC, ref. #94770; PubChem CID: 8468). All reagents were dissolved in dimethyl sulfoxide (DMSO).

### 3.2. Cell Culture

Mouse 3T3-L1 preadipocytes (#CL-173^TM^, American Type Culture Collection, ATCC, Manassas, VA, USA) were grown in complete Dulbecco’s modified Eagle’s medium (DMEM, Invitrogen, Carlsbad, CA, USA) supplemented with 10% calf blood serum (CBS) and 1% penicillin/streptomycin at 37 °C, 5% CO_2_ and 95% humidity until performing the evaluations.

### 3.3. Cell Viability Assay

The influence of the phenolic compounds on 3T3-L1 cellular viability was assessed by the MTS assay. For the cytotoxicity assay, 3T3-L1 preadipocytes were plated in triplicate into 96 well plates (5 × 10^3^ cells/well) and incubated in complete medium supplemented with each compound at different doses (1, 10, 50, 100, 250 µM) for 48 h. MTS solution (CellTiter 96^®^ AQueous One Solution Cell Proliferation Assay, Promega, Madison, WI, USA) was added to the cells and incubated for three h at 37 °C. The cell viability was then evaluated through the colorimetric quantification of MTS at 492 nm using a microplate spectrophotometer (Thermo Scientific Multiskan; Thermo Fisher Scientific Inc., Waltham, MA, USA).

### 3.4. Effect of Phenolic Compounds on Adipogenesis

To evaluate the effect of phenolic compounds on adipogenesis, 3T3-L1 cells were cultured in 96 (5 × 10^3^ cells/well) or in 12 well (7 × 10^4^ cells per well) plates for the quantification of triglyceride levels (Nile Red staining) and for gene expression, respectively [61]. On the second day post-confluence (d0) adipocyte differentiation was induced by incubating the cells in DMEM supplemented for 48 h with “MDI differentiation cocktail”, constituted by 10% foetal bovine serum (FBS), 3-isobutyl-1-methylxanthine (IBMX) (0.5 mM), dexamethasone (1 mM) and insulin (1 mg/mL). The medium was then replaced by DMEM containing 10% foetal bovine serum (FBS) and insulin (1 mg/mL) and incubated for 72 h, until day 5 (d5). Cells were maintained in DMEM containing 10% foetal bovine serum (FBS) until day 8 (d8), day 13 (d13) and day 16 (d16), when 96 well plates were stained with Nile Red (S1, S2 and S3 assays, respectively) and adipocytes from 12 well plates were harvested for gene expression analyses. Undifferentiated cells (as control of 3T3-L1 preadipocyte stage) were maintained during all differentiation (from day 0 to day 16) in DMEM supplemented with 10% of calf-bovine serum (CBS). The effect of polyphenols and phenolic acids on adipogenesis was evaluated at three different times of adipocyte differentiation, depending on the moment the compounds were added to the medium (Figure 1): pre-differentiation (S1, compounds added on day 0 and cells collected on day 8), differentiation (S2, compounds added on day 5 and cells collected on day 13) and post-differentiation (S3, compounds added on day 8 and cells collected on day 16). Undifferentiated 3T3-L1 cells were included as negative control.

Three different doses (10, 50, 100 µM) were evaluated in each case and compounds were maintained in the medium for 8 days, when cells were collected for triglyceride quantification and gene expression analysis. For all conditions, DMSO was added at concentrations lower than 0.1%.

### 3.5. Intracellular Triglyceride Quantification

Intracellular triglyceride content was quantified (Nile Red staining) to estimate the degree of cell differentiation [61,62,63]. 3T3-L1 preadipocytes (day 0), immature adipocytes (day 5) and mature adipocytes (day 8) were cultured in 96 well plates together with the phenolic compounds as described previously, using 8 replicates for each condition. After treatments, cells were washed three times with phosphate buffered saline (PBS) and fixed with formaldehyde 3.7% for 30 min. Then, cells were washed with isopropanol 60% for 10 min and, after drying, incubated for 30 min with Nile Red (5 µg/mL). Cells were washed three times with PBS and the fluorescence was quantified with a fluorometer (BMG Lab Tech POLARstar OPTIMA Fluorometer, BioSurplus, San Diego, CA, USA) using 485 nm excitation and 520 nm emission filters. The degree of lipid accumulation was calculated as the relative fluorescent emission of each sample relative to the control (0.1% DMSO). For all treatments, results from at least three independent experiments were considered.

### 3.6. Cohen’s D Effect Size Calculation

The effect of the different treatments on 3T3-L1 differentiation (measured by Nile Red) was classified by Cohen’s D effect size [64,65], using the following equations [64]:

D = (M1 − M2)/σp(1)

where M1 and M2 are the evaluated effects and σp corresponds to their pooled standard deviation, calculated with the formula:

σp = √[(σ12 + σ22)/2](2)

### 3.7. Gene Expression Quantification (qPCR)

3T3-L1 preadipocytes were cultured in 12 well plates in the presence of the MDI differentiation cocktail, together with 20 µM of each phenolic compound. The experiment was performed as previously indicated for S1 pre-differentiation assay. On day 8, total RNA from 3T3-L1 adipocytes were extracted using Trizol^®^ RNA isolation reagent (Thermo Fisher Scientific Inc.) according to the manufacturer’s instructions. Concentration and purity of RNA were determined at 260/280 nm using a NanoDrop spectrophotometer (Thermo Fisher Scientific Inc.). Then, 500 ng of RNA were treated with DNAse (Ambion™ DNase I, RNase-free; Thermo Fisher Scientific Inc.) according the manufacturer’s protocol. For retrotranscription into cDNA, 50 µm of dNTPs mix (Bioline, Luckenwalde, Germany) and 25 ng/µL of Random Primers (Invitrogen) were added to the RNA and incubated 5 min at 65 °C. Afterwards, 40 units of recombinant RNAsin^®^ Ribonuclease inhibitor (Promega, Madison, WI, USA), 5 mM of dithiothreitol (DTT, Invitrogen) and the First Strand Buffer (Invitrogen) were added and samples incubated 2 min at 37 °C. Finally, 200 units of M-MLV reverse transcriptase (Invitrogen) were added to each tube and incubated for 10 minutes at 25 °C, 50 min at 37 °C and 15 min at 70 °C.

Gene expression analyses were performed by quantitative-real time PCR (qPCR) using the TaqMan Universal PCR master mix and specific probes (Table S1) from Applied Biosystems Technologies (Thermo Fisher Scientific Inc.) and Integrated DNA Technologies Inc., (Coralville, IA, USA). All reactions were performed using a CFX384 Touch™ Real-Time PCR Detection System (BioRad, Hercules, CA, USA). The expression levels of each gene were normalised against the TATA box binding protein gene (*Tbp*) as housekeeping gene control. Gene expression differences between treated and untreated samples were quantified using the relative quantification 2^−∆∆Ct^ method [66].

### 3.8. Molecular Docking Analysis: DockingServer

In order to investigate the potential role of phenolic compounds as effectors through their putative interaction with PPARγ protein, molecular docking was calculated using DockingServer (www.dockingserver.com) [45,46]. The three-dimensional (3D) coordinates of the crystal structure of PPARγ were obtained from Protein Data Bank (PDB) (sequence 1PRG) [9,31], selecting both chains (α and β) for the analysis, using Gasteiger charge calculation method and adding partial charges to the ligand atoms [67]. Box size was of 100 Å for x, y and z axis. Non-polar hydrogen atoms were merged, and rotatable bonds were defined. 3D structures of all phenolic compounds were downloaded in SDF format from PubChem database [68] and transformed into MOL2 format using Openbabel [69]. Essential hydrogen atoms, Kollman united atom type charges, and solvation parameters were added using AutoDock tools [70]. AutoDock parameter set- and distance-dependent dielectric functions were used in the calculation of the van der Waals and the electrostatic terms, respectively. Initial position, orientation, and torsions of the ligand molecules were set randomly. All rotatable torsions were released during docking. Each docking experiment was derived from 10 different runs that were set to terminate after a maximum of 250,000 energy evaluations. The population size was set to 150. During the search, a translational step of 0.2 Å, and quaternion and torsion steps of 5 were applied.

### 3.9. Statistical Analyses

For the assays of differentiation, statistical analysis was performed using 2-way Anova, followed by the Student-Newman-Keuls (SNK) test. In the assays of cytoxicity, results were evaluated through non-parametric statistics using a Kruskal-Wallis test, followed by a Wilcoxon test comparing each group to the control when significant differences were obtained. Real-time PCR data was analysed using Student T test comparing each treatment to its control. Correlation analysis was performed using the Pearson correlation coefficient. All statistical analyses were performed using STATA software (Stata v12; StataCorp, LLC, College Station, TX, USA).

## 4. Conclusions

Polyphenols and phenolic acids show different anti-adipogenic activity on the 3T3-L1 preadipocyte model depending on the stage of the differentiation process. Quercetin, resveratrol and apigenin are the most effective polyphenols, while vanillic was the most efficient phenolic acid. Resveratrol and quercetin inhibit the whole process of differentiation, while apigenin and myricetin reduce the triglyceride content in the early and late stages of differentiation, but not in the intermediate. On the contrary, hesperidin exhibits a specific inhibitory activity focused to the intermediate stages of the differentiation process. With the exception of *p*-coumaric acid, phenolic acids strongly inhibit the early stages of adipogenesis, but their anti-lipogenic activity is low in mature adipocytes. The anti-adipogenic effect of the majority of these phenolic compounds is associated with their capacity to down-regulate the expression of adipocyte-specific genes, such as *Scd1* and *Lpl*.

Molecular docking results suggest that polyphenols and phenolic acids may inhibit the early stages of adipogenesis by interacting with PPARγ receptor, providing a mechanistic insight into possible pharmacological effects ant therapeutic benefits. This binding affinity to PPARγ is associated with some amino acids located into the binding pocket of the receptor, mainly residues Phe264, His266, Ile281, Cys285 and Met348, which might play a crucial role modulating the activity of this transcription factor. The qPCR analysis of PPARγ-target genes (*Adipoq*, *Plin1* and *Fabp4*) would support the modulating effect of these compounds on this receptor. 

In conclusion, treatment with polyphenols and phenolic acids might exert beneficial effects on lipid metabolism by attenuating adipogenic differentiation through their interaction with PPARγ. Further research on this interaction might be necessary to elucidate the in vivo mechanisms of these molecules as potential therapeutic targets to prevent obesity and related diseases.

## Figures and Tables

**Table 1:** Free energy values for the ligand-protein interactions predicted by DockingServer. Troglitazone and aglycones from Naringin and Hesperidin (Naringenin and Hesperetin) are shown, shadowed in grey, for comparative purposes.

	Estimated Free Energy of Binding (kcal/mol)	Estimated Inhibition Constant, Ki (uM)	vdW + Hbond + desolv Energy (kcal/mol)	Total Intermolec. Energy (kcal/mol)	Interaction Surface
Troglitazone	−7.23	5.00	−8.83	−8.93	1021.969
Hesperetin	−5.95	43.34	−6.38	−6.42	942.649
Luteolin	−5.84	52.07	−6.07	−6.32	667.019
Apigenin	−5.78	57.62	−6.30	−6.32	774.163
Naringenin	−5.77	58.80	−6.27	−6.30	897.003
Ellagic acid	−5.64	73.75	−5.59	−5.70	871.940
Kaempferol	−5.27	137.56	−5.67	−5.89	902.514
Quercetin	−5.07	193.00	−5.64	−5.85	672.588
Resveratrol	−4.86	273.59	−6.04	−6.36	614.078
Curcumin	−4.86	272.69	−6.70	−6.95	1052.665
*p*-Coumaric acid	−4.44	558.07	−5.39	−5.64	451.466
Vanillic acid	−4.40	591.18	−4.99	−5.24	427.083
Myricetin	−4.34	654.56	−4.84	−5.01	724.325
Gallic acid	−4.05	1080	−4.48	−4.76	407.548
Ferulic acid	−4.00	1180	−5.04	−5.11	567.571
Naringin	−3.98	1200	−5.84	−5.85	657.646
Hesperidin	−3.16	4810	−4.94	−5.07	561.726

**Table 2:** Common amino acids of the ligand-binding domain (LBD) of PPARγ implicated in the interactions with the different phenolic compounds, indicated by black dots (●).

	Troglitazone	Quercetin	Resveratrol	*p*-coumaric acid	Luteolin	Myricetin	Kaempferol	Curcumin	Ellagic acid	Apigenin	Ferulic acid	Gallic acid	Vanillic acid	Hesperetin *	Naringenin *
Leu^228^	●							●							
Leu^255^			●	●	●	●									
Glu^259^		●	●	●	●										
Lys^263^						●									
Phe^264^		●	●	●	●	●			●					●	
His^266^	●	●	●	●	●									●	
Arg^280^		●	●	●	●										●
Ile^281^		●	●	●	●				●						●
Cys^285^		●	●	●	●	●	●	●	●	●					
Arg^288^	●				●		●	●		●					●
Ser^289^							●				●				
Glu^291^	●							●	●	●				●	●
Glu^295^							●	●							
Ile^326^										●	●				
Tyr^327^										●	●				
Leu^330^										●	●			●	
Leu^333^	●						●	●		●	●			●	●
Val^339^										●				●	
Ile^341^		●	●	●			●							●	●
Met^348^		●	●	●	●	●			●						
Phe^363^										●	●				
Glu^418^												●	●		
Leu^421^												●	●		
Ser^428^												●	●		
Leu^431^												●	●		
Phe^432^												●	●		
His^449^										●	●				

* Naringin and hesperidin were excluded from the table due to they do not share any common amino acids in their binding with the receptor. Their respective aglycones (naringenin and hesperetin) are shown, shadowed in grey, for comparative purposes.

## Supplementary Materials

Supplementary tables and figures are available online. Figure S1, Images obtained from observation under a fluorescence microscope of the effect of the different treatments in 3T3-L1 after Nile Red staining. The figure corresponds to the effect of the treatments at the dose of 100 uM and the S1 test. (**A**) Triglyceride content of 3T3-L1 adipocytes after treatment with polyphenols. (**B**) Triglyceride content of 3T3-L1 adipocytes after treatment with phenolic acids. Figure S2, Structures of the phenolic compounds evaluated in this study. The hydrogens indicated with the symbol * are involved in the interactions with the LBD of PPARγ. Table S1, TaqMan gene expression assays used for the quantitative real time PCR analysis. Table S2, Quantification of the toxicity induced by phenolic compounds in 3T3-L1 preadipocytes. Results are expressed as the mean cell viability in percentage (±SD) relative to DMSO-treated control cells. Shadowed are the concentrations at which a significant decrease in cell viability is observed. Table S3, Cohen’s D effect size of the anti-adipogenic activity (quantified by Nile Red) for each phenolic compound during S1, S2 and S3 stages. Table S4, Interactions of each phenolic compound with the specific amino acids of the PPARγ LBD, predicted by DockingServer (H for hydrogen, C for carbon and O for oxygen). Troglitazone and aglycones from naringin and hesperidin (naringenin and hesperetin) are shown shadowed for comparative purposes.
