# Supplementary material for: Phenolic Compounds Inhibit 3T3-L1 Adipogenesis Depending on the Stage of Differentiation and Their Binding Affinity to PPARγ
Source: Molecules. 2019 Mar 16;24(6):1045. doi: 10.3390/molecules24061045 (PMC6470710; doi:10.3390/molecules24061045)
Supplement: Supplementary file 1 [file molecules-24-01045-s001.pdf]

Supplementary Tables

Table S1. TaqMan gene expression assays used for the quantitative real time PCR analysis.

GENE	DESCRIPTION	Gene Expression Assay reference
Pparg	Peroxisome proliferator activated receptor gamma	Mm00440940_m1*
Cebpa	CCAAT/enhancer binding protein (C/EBP), alpha	Mm00514283_s1*
Fasn	Fatty acid synthase	Mm00662319_m1*
Scd1	Stearoyl-Coenzyme A desaturase 1	Mm00772290_m1*
Lpl	Lipoprotein lipase	Mm00434764_m1*
Tbp	TATA box binding protein. Housekeeping gene control	Mm00446973_m1*
Adipoq	Adiponectin	Mm.PT.58.9719546 **
Plin1	Perilipin 1	Mm.PT.58.10000337 **
Fabp4	Fatty acid binding protein 4, adipocyte	Mm.PT.58.43866459**

* TaqMan® Gene Expression Assays (Thermo Fisher Scientific).

** PrimeTime qPCR Probe Assays (Integrated DNA Technologies, Inc).

Table S2. Quantification of the toxicity induced by phenolic compounds in 3T3-L1 preadipocytes. Results are expressed as the mean cell viability in percentage (±SD) relative to DMSO-treated control cells. Shadowed are the concentrations at which a significant decrease in cell viability is observed.

	**1 μM**	**10 μM**	**50 μM**	**100 μM**	**250 μM**
Hesperidin	102.89 ± 1.59	95.70 ± 4.59	92.55 ± 9.84	102.09 ± 1.95	54.02 ± 9.34 ***
Naringin	89.00 ± 17.40	59.58 ± 4.65 **	55.19 ± 11.73 **	64.13 ± 10.05 **	39.25 ± 25.57 ***
Apigenin	104.39 ± 5.57	104.32 ± 2.37	102.66 ± 1.84	86.67 ± 7.54 **	33.28 ± 2.74 ***
Luteolin	78.41 ± 19.61 *	68.62 ± 16.32 **	60.70 ± 15.04 **	32.60 ± 8.57 ***	24.77 ± 10.02 ***
Myricetin	111.67 ± 3.91	104.18 ± 5.61	98.38 ± 13.19	54.92 ± 1.46 ***	59.92 ± 10.64 ***
Kaempferol	75.64 ± 4.38 *	28.64 ± 5.63 ***	48.69 ± 11.31 ***	64.91 ± 20.57 **	68.38 ± 1.98 **
Quercetin	111.33 ± 0.43	112.99 ± 1.07	118.76 ± 3.92 *	122.36 ± 2.42 *	116.21 ± 15.59
Resveratrol	92.64 ± 10.53	106.55 ± 6.08	92.12 ± 5.42	64.16 ± 6.16 ***	47.36 ± 9.64 ***
Curcumin	93.07 ± 2.39	94.87 ± 1.93	47.82 ± 19.41 #	36.35 ± 3.99 *	37.39 ± 0.47 **
p-Coumaric acid	99.49 ± 1.18	100.27 ± 2.37	99.61 ± 4.18	101.37 ± 2.49	101.02 ± 5.48
Ellagic acid	102.16 ± 4.99	103.65 ± 1.27	96.00 ± 0.1	99.25 ± 2.61	107.88 ± 2.16
Ferulic acid	103.24 ± 5.63	100.27 ± 3.52	106.83 ± 4.18	101.89 ± 1.57	97.66 ± 3.82
Gallic acid	102.29 ± 4.59	106.02 ± 4.08	99.82 ± 1.91	101.35 ± 2.49	104.09 ± 3.74
Vanillic acid	95.37 ± 2.71	99.25 ± 4.52	107.18 ± 4.61 *	97.88 ± 7.36	93.55 ± 1.23

Table S3. Cohen's D effect size of the anti-adipogenic activity (quantified by Nile Red) for each phenolic compound during S1, S2 and S3 stages.

	NR S1	NR S2	NR S3
**3T3-L1 preadipocytes**	12.52	11.57	17.02
**3T3-L1 DMSO control**	0.00	0.00	0.00
**Hesperidin**	-0.20	1.00	0.48
**Myricetin**	1.95	0.32	1.87
**Quercetin**	5.32	5.27	4.34
**Apigenin**	2.44	0.38	2.57
**Resveratrol**	3.29	4.34	1.76
**Curcumin**	0.78	0.00	1.05
***p*-Coumaric Acid**	1.48	0.57	1.53
**Ellagic acid**	2.34	2.10	-0.50
**Ferulic acid**	2.14	0.76	-0.21
**Galic acid**	3.16	0.00	0.66
**Vanillic acid**	4.35	1.02	-0.08

Table S4. Interactions of each phenolic compound with the specific amino acids of the PPARγ LBD, predicted by DockingServer (H for hydrogen, C for carbon and O for oxygen). Troglitazone and aglycones from naringin and hesperidin (naringenin and hesperetin, respectively) are shown shadowed for comparative purposes.

	Hydrogen bonds	Polar	Cation-pi	Hydrophobic	pi-pi	Other
Troglitazone	O-LYS265 (N)[3.38]			C-HIS266 (CD2) [3.51]<br>C-LEU228 (CD2) [3.74]<br>C-LEU333 (CD1) [3.89]<br>C-PHE287 (CD1) [3.75]		O-LEU228 (CB,CD2) [3.39]<br>N-LEU228 (CD2) [3.43]<br>H-LEU228 (CD2) [2.89]<br>C-HIS266 (NE2) [3.49]<br>O-PHE287 (CD1) [3.89]<br>C-ARG288 (CG) [3.78]<br>C-GLU291 (CD,OE1,OE2) [2.85]<br>S-GLU291 (OE2) [3.62]<br>H-LEU333 (CD1) [3.84]<br>O-LEU333 (CD1) [3.76]
Hesperetin		O-GLU291 (OE1) [3.62]<br>H-GLU291 (OE1, OE2) [3.29]	H-PHE264 (CB, CD1, CG) [3.49]<br>H-HIS266 (CD2, CG) [2.87]	C-HIS266 (CD2) [3.64]<br>C-LEU333 (CD1) [3.60]<br>C-ILE341 (CB,CG2) [3.56]		O-PHE264 (CB, CD1, CG) [3.65]<br>O-HIS266 (CD2) [3.35]<br>H-GLU291 (CG) [3.67]<br>O-LEU330 (CD1) [3.78]<br>O-LEU333 (CD1) [3.39]<br>H-LEU333 (CD1) [3.47]<br>O-VAL339 (CG2) [3.67]<br>H-VAL339 (CG2) [2.95]<br>O-ILE341 (CB,CG2) [3.60]
Luteolin	O-ARG280 (*CB, NH2*) [3.42]<br>O-CYS285 (*SG*) [3.29]	O-GLU259 (OE2) [3.25]<br>H-GLU259 (OE2) [2.29]<br>H-ARG280 (CZ, NE, NH2) [3.36]<br>O-ARG288 (CB) [3.53]		C-LEU255 (CD1) [3.80]<br>C-CYS285 (SG) [3.82]<br>C-ILE341 (CG2) [3.86]	C-PHE264 (CB, CD2, CG) [3.31]<br>C-HIS266 (CB, CD2, CG) [3.06]	H-LEU255 (CD1, CD2, CG) [2.94]<br>O-LEU255 (CD1, CD2) [3.37]<br>H -GLU259 (CD) [3.33]<br>C-GLU259 (OE2) [3.67]<br>O-HIS266 (CB) [3.88]<br>O-ILE281 (CG1) [3.60]<br>H-CYS285 (SG) [2.57]<br>C-ARG288 (CB) [3.87]<br>O-ILE341 (CG2) [3.50]

	Hydrogen bonds	Polar	Cation-pi	Hydrophobic	pi-pi	Other
Apigenin	O-TYR327 (CE1,CZ,OH) [3.06]	O-ARG288 (CB, NH2) [3.56]	H-TYR327 (CE1, CZ) [2.73]	C-CYS285 (CB, SG) [3.51]	C-TYR327 (CE1) [3.40]	C-ARG288 (CB, NE, NH2) [3.23]
		O-GLU291 (CE1) [3.90]	H- HIS449 (CD2) [3.42]	C-ILE326 (CG2) [3.32]	C- PHE363 (CZ) [3.83]	H-GLU291 (CB) [3.29]
		H-GLU291 (CE1) [3.58]		C-LEU330 (CG) [3.90]		H-ALA292 (CB) [3.82]
		H-TYR327 (OH) [2.22]		C-LEU333 (CD1) [3.83]		O-LEU333 (CD1) [3.33]
		O-HIS449 (NE2) [3.44]		C-MET364 (SD) [3.69]		H-LEU333 (CD1) [3.48]
		H-HIS449 (NE2) [3.26]				O-VAL339 (CG2) [3.78]
						O-PHE363 (CE1,CZ) [3.37]
						O-HIS449 (CD2) [3.66]

Table S4 (cont). Interactions of each phenolic compound with the specific amino acids of the PPARγ LBD, predicted by DockingServer (H for hydrogen, C for carbon and O for oxygen). Troglitazone and aglycones from naringin and hesperidin (naringenin and hesperetin, respectively) are shown shadowed for comparative purposes.

	Hydrogen bonds	Polar	Cation-pi	Hydrophobic	pi-pi	Other
Naringenin		H-ARG280 (CB) [3.88]		C-ILE341 (CB,CG2) [3.52]		H-ILE281 (CG1) [3.89]
		O-GLU291 (OE1,OE2) [3.51]				C-ARG288 (CG) [3.78]
		H-GLU291 (OE1,OE2) [2.95]				H-GLU291 (CD) [3.72]
						O-LEU333 (CD1) [3.76]
						H-LEU333 (CD1) [3.47]
Ellagic acid		O-ARG288 (CB,CG) [3.58]				O-PHE264 (CD1) [3.77]
		O-GLU291 (CE1,CE2) [3.17]				H-ILE281 (CB, CG1, CG2) [3.39]
		H-GLU291 (CE1,CE2) [2.26]				O-ILE281 (CG2) [3.39]
						O-CYS285 (SG) [3.80]
						H-CYS285 (SG) [3.04]
						C-ARG288 (CB,CG) [3.43]
						O-GLU291 (CD) [3.78]
						H-GLU291 (CD) [2.95]
						O-MET348 (CE) [3.63]

Kaempferol	O-CYS285 (SG) [3.62]	O-ARG288 (CB, NH2) [3.49]<br>H-SER289 (OG) [3.68]<br>H-GLU295 (CE1,CE2) [1.93]<br>O-GLU295 (OE2) [2.89]	C-CYS285 (CB,SG) [3.73]<br>C-LEU333 (CD1) [3.68]	O-PRO227 (CB, CD, CG) [3.25]<br>H-PRO227 (CB, CD, CG) [2.93]<br>H-CYS285 (CB) [3.72]<br>O-ARG288 (CB, NH2) [3.26]<br>O-SER289 (CB) [3.40]<br>H-SER289 (CB) [2.96]<br>O-GLU295 (CD) [3.79]<br>H-GLU295 (CD) [2.87]<br>C-GLU295 (OE2) [3.61]<br>O-LEU333 (CD1) [3.68]<br>H-LEU333 (CD1) [3.86]<br>O-ILE341 (CD1,CG1) [3.39]<br>H-ILE341 (CD1,CG1) [3.27]

Table S4 (cont). Interactions of each phenolic compound with the specific amino acids of the PPARγ LBD, predicted by DockingServer (H for hydrogen, C for carbon and O for oxygen).

	Hydrogen bonds	Polar	Cation-pi	Hydrophobic	pi-pi	Other
Quercetin	O-ARG280 (NE,NH2) [3.40]	O-GLU259 (OE2) [3.56]	H-PHE264 (CB) [3.61]	C-ILE281 (CG1) [3.67]	C-PHE264 (CD2) [3.66]	O-GLU259 (CD) [3.89]
		H-GLU259 (OE2) [2.93]	H-HIS266 (CB, CD2, CG) [3.07]	C-CYS285 (SG) [3.27]	C-HIS266 (CB) [3.78]	H-GLU259 (CD) [3.43]
		H-ARG280 (NE,NH2) [3.68]		C-ILE341 (CB,CG2) [3.21]		O-PHE264 (CB,CD2,CG) [3.21]
				C-MET348 (CE,SD) [3.19]		O-HIS266 (CB) [3.61]
						C-ARG280 (CB) [3.64]
						O-CYS285 (SG) [3.43]
						H-CYS285 (SG) [3.19]
						H-ILE341 (CB) [3.61]
						O-MET348 (CE) [3.32]
						H-LEU353 (CD1,CD2) [3.75]
Curcumin		O-ARG288 (CB, NH2) [3.40]		C-LEU333 (CD1) [3.51]		O-LEU228 (CB) [3.41]
		O-GLU343 (OE1) [3.69]				O-CYS285 (SG) [3.81]
						H-CYS285 (SG) [3.39]
						C-ARG288 (CB,NE,NH2) [3.44]
						C-GLU291 (CB,CD,CG,OE2) [3.23]
						C-GLN294 (CB) [3.84]
						C-GLU295 (CD,CG,OE1,OE2) [3.07]
						C-GLU343 (OE1) [3.61]
Resveratrol	O-ARG280 (NE,NH2) [3.30]	O-GLU259 (OE2) [3.04]	H-HIS266 (CB) [3.70]	C-LEU255 (CD1) [3.54]	C-PHE264 (CD2) [3.52]	C-GLU259 (OE2) [3.90]
		H-GLU259 (OE1,OE2) [2.10]		C-ILE281 (CG1) [3.81]	C-HIS266 (CB) [3.48]	H-GLU259 (CD) [3.11]
		O-ARG288 (CB) [3.48]		C-CYS285 (SG) [3.66]		O- HIS266 (CB) [3.54]
		H-ARG288 (CB) [3.05]		C-ILE341 (CG2) [3.49]		C-ARG280 (CB,NE) [3.51]
				C-MET348 (CE) [3.09]		O-CYS285 (SG) [3.47]
						H-CYS285 (SG) [2.66]

						O-ILE341 (CG2) [3.60]
						O-MET348 (CE,SD) [3.63]
p-Coumaric acid		O-GLU259 (OE2) [3.00]		C-LEU255 (CD1,CD2) [3.50]		O-LEU255 (CD1) [3.79]
		H-GLU259 (OE2) [2.18]		C-PHE264 (CB,CD2,CG) [3.48]		H-LEU255 (CD1) [3.77]
		O-ARG280 (NH2) [3.46]		C-HIS266 (CB) [3.73]		H-GLU259 (CD,CG) [3.18]
				C-ILE281 (CG1) [3.75]		H-ILE281 (CB,CG2) [3.24]
				C-MET348 (CE) [3.49]		O-CYS285 (SG) [3.88]
						H-CYS285 (SG) [3.84]
						O-CYS285 (SG) [3.47]
						O-ILE341 (CG2) [3.69]
						O-MET348 (CE) [3.71]

Table S4 (cont). Interactions of each phenolic compound with the specific amino acids of the PPARγ LBD, predicted by DockingServer (H for hydrogen, C for carbon and O for oxygen).

	**Hydrogen bonds**	**Polar**	**Cation-pi**	**Hydrophobic**	**pi-pi**	**Other**
Vanillic acid		O-GLU418 (OE2) [3.07]		C-LEU421 (CB) [3.56]		O-GLU418 (CD, CG) [3.58]
		H-GLU418 (OE1,OE2) [2.11]		C-PHE432 (CB) [3.20]		O-LEU421 (CB) [3.81]
						O-LYS422 (CE) [3.63]
						H-SER428 (CB) [3.75]
						O-LEU431 (CB) [3.21]
						H-LEU431 (CB) [3.45]
						H-GLU418 (CD) [3.10]
						C-GLU418 (OE2) [3.76]
Myricetin	O-LYS263 (CD,CE,CG,NZ) [3.07]	H-LYS263 (NZ) [2.64]		C-MET348 (CE) [3.60]	C-PHE264 (CD1,CE1) [3.46]	O-LEU255 (CD1) [3.82]
		O-GLN345 (NE2) [3.40]				H-LEU255 (CD1,CD2) [2.88]
		H-GLN345 (NE2) [3.19]				H-LYS263 (CD,CG) [3.49]
						O-PHE264 (CD1,CE1,CG) [3.48]

						H-CYS285 (SG) [3.67]
						C-SER342 (CB) [3.19]
						O-SER342 (CB) [3.29]
						H-SER342 (CB) [2.73]
						O-MET348 (CE) [3.68]
Gallic acid		O-GLU418 (OE2) [3.49]	H-PHE432 (CD2) [3.79]	C-PHE432 (CB) [3.52]		O-GLU418 (CG) [3.63]
		H-GLU418 (OE2) [2.83]				H-GLU418 (CD,CG) [3.72]
		O-GLN430 (NE2) [3.16]				O-LEU421 (CB) [3.83]
						H-SER428 (CB) [3.58]
						O-LEU431 (CB) [3.23]
						H-LEU431 (CB) [3.28]
						O-PHE432 (CB) [3.44]
Ferullic acid	O-GLN286 (CD,NE2,OE1) [3.17]	H-TYR327 (OH) [2.14]	H-TYR327 (CE1,CZ) [2.99]	C-CYS285 (CB,SG) [3.69]		C-SER289 (CB) [3.84]
	O-TYR327 (CE1,CZ,OH) [3.05]	O-HIS449 (NE2) [3.60]	H-HIS449 (CD2,CE1) [3.68]	C-ILE326 (CG2) [3.71]		O-LEU330 (CD2,CG) [3.80]
		H-HIS449 (NE2) [3.26]		C-LEU330 (CB,CG) [3.56]		H-LEU330 (CD2) [3.90]
						H-LEU330 (CD1) [3.12]
						O-PHE363 (CZ) [3.78]

Table S4 (cont). Interactions of each phenolic compound with the specific amino acids of the PPARγ LBD, predicted by DockingServer (H for hydrogen, C for carbon and O for oxygen).

	Hydrogen bonds	Polar	Cation-pi	Hydrophobic	pi-pi	Other
Naringin	O-SER355 (OG) [2.96]	O-GLU272 (OE1) [3.65]	H-PHE352 (CD1,CE1) [3.20]	C-MET252 (CG) [3.40]		H-ASP251 (CB) [3.36]
		H-GLU272 (OE1) [3.68]				O-ASP251 (CB) [3.77]
		O-GLU276 (OE1) [3.49]				C-GLU272 (OE1,OE2) [3.43]
		H-GLU276 (OE1) [2.70]				H-GLU276 (CD) [3.65]
		H-SER355 (OG) [2.02]				C-GLU276 (OE1) [3.17]
		O-ARG357 (NE) [3.84]				O-PHE352 (CD1,CE1) [3.19]
		H-ARG357 (CZ,NE,NH2) [3.24]				H-SER355 (CB) [3.21]
						C-SER355 (OG) [3.13]
						C-ARG357 (CB, CG) [3.60]
						C-LYS358 (CD,CE,CG) [3.62]
						O-LYS358 (CD) [3.38]
Hesperidin	O-ASN412 (CB,ND2) [3.43]	ARG357 (*CZ, NE, NH2*)		C-LEU309 (CD2) [3.88]		O-ASN308 (CB) [3.34]
				C-PRO405 (CB) [3.18]		H-ASN308 (CB) [3.24]
				C-ILE409 (CD1,CG1) [3.18]		O-LEU309 (CD2) [3.54]
						O-PRO405 (CB) [3.88]
						C-ASP408 (CB) [3.68]
						H-ASN412 (CB,CG) [3.37]
						C-ASN412 (ND2) [3.79]

Supplementary Figures

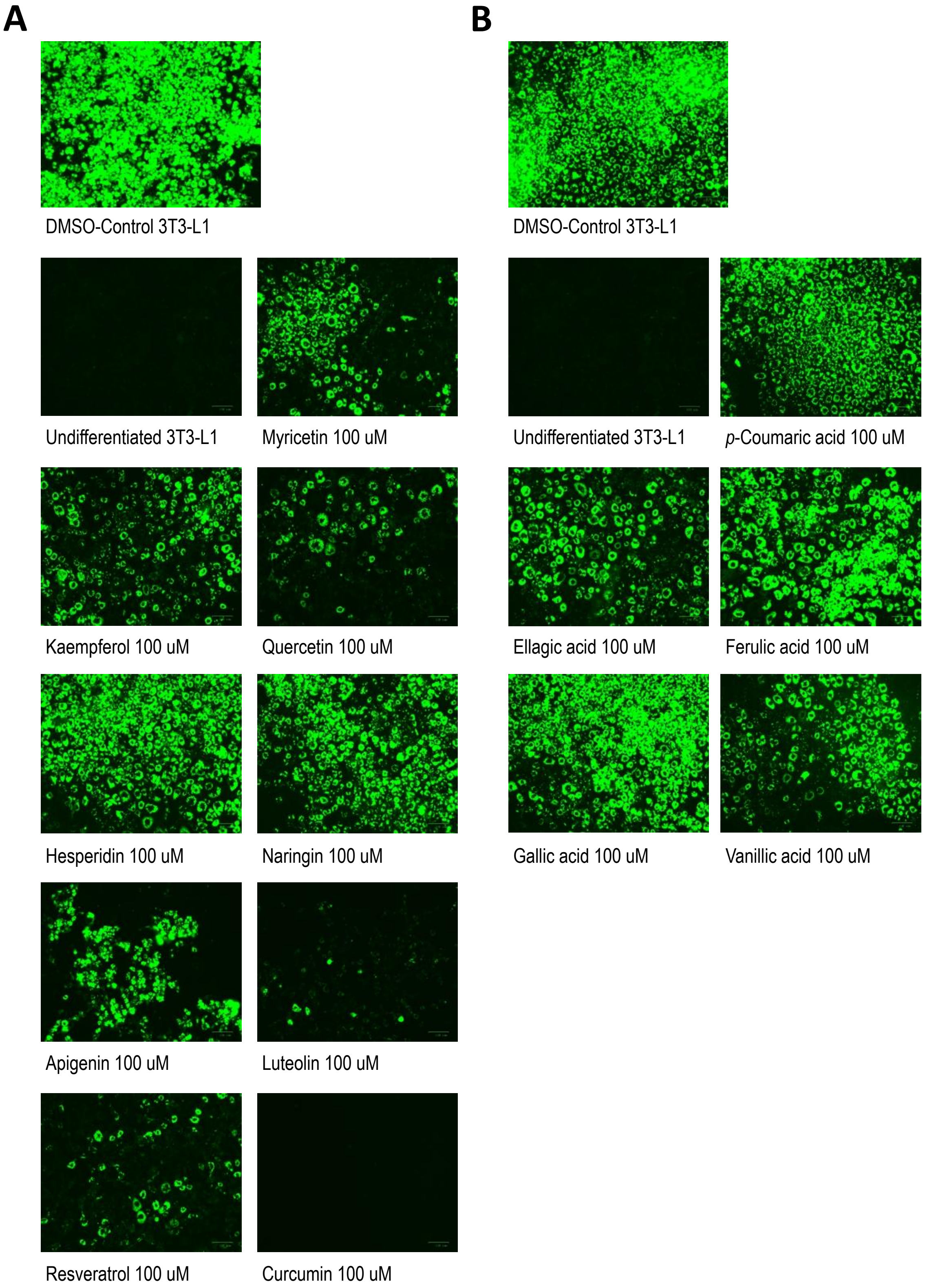

Figure S1. Images obtained from observation under a fluorescence microscope of the effect of the different treatments in 3T3-L1 after Nile Red staining. The figure corresponds to the effect of the treatments at the dose of 100 uM and the S1 test. A) Triglyceride content of 3T3-L1 adipocytes after

treatment with polyphenols. B) Triglyceride content of 3T3-L1 adipocytes after treatment with phenolic acids.

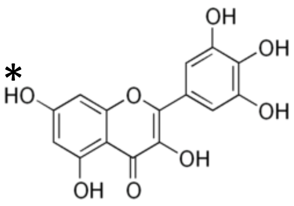

Myricetin

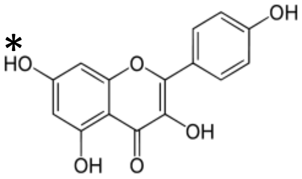

Kaempferol

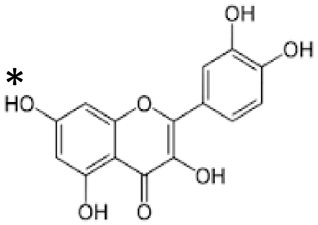

Quercetin

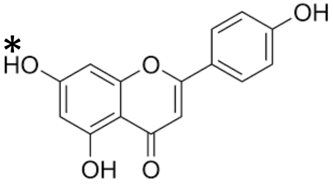

Apigenin

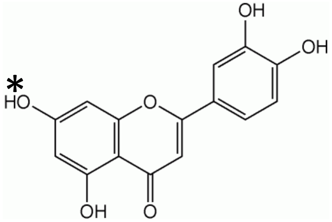

Luteolin

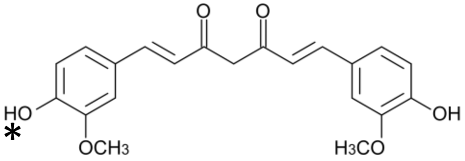

Curcumin

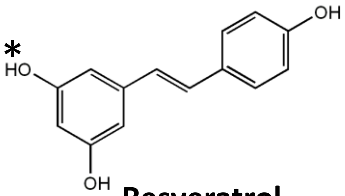

Resveratrol

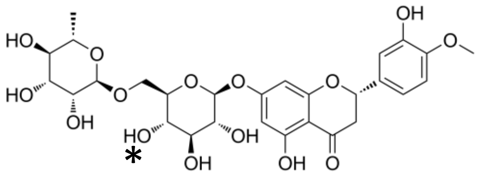

Hesperidin

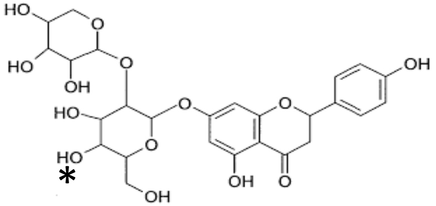

Naringin

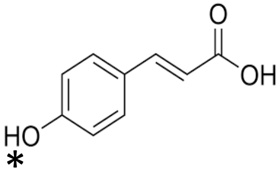

***p*-Coumaric acid**

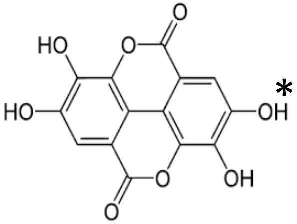

Ellagic acid

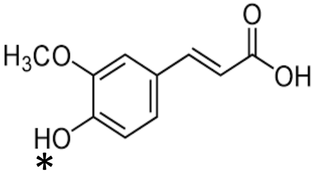

Ferulic acid

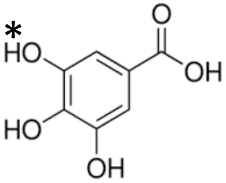

Gallic acid

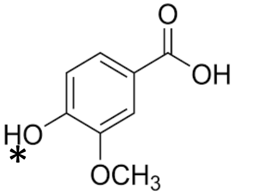

Vanillic acid

Figure S2. Structures of the phenolic compounds evaluated in this study. The hydrogens indicated with the symbol * are involved in the interactions with the

LBD of PPARγ.
